# An Adenosine A_2A_ Receptor Antagonist Improves Multiple Symptoms of Repeated Quinpirole-Induced Psychosis

**DOI:** 10.1523/ENEURO.0366-18.2019

**Published:** 2019-02-27

**Authors:** Nozomi Asaoka, Naoya Nishitani, Haruko Kinoshita, Yuma Nagai, Hikari Hatakama, Kazuki Nagayasu, Hisashi Shirakawa, Takayuki Nakagawa, Shuji Kaneko

**Affiliations:** 1 Department of Molecular Pharmacology, Graduate School of Pharmaceutical Sciences, Kyoto University, Sakyo-ku, Kyoto 606-8501, Japan; 2 Department of Clinical Pharmacology and Therapeutics, Kyoto University Hospital, Sakyo-ku, Kyoto 606-8507, Japan

**Keywords:** D_2_ receptor, obsessive-compulsive disorder

## Abstract

Obsessive-compulsive disorder (OCD) is a neuropsychiatric disorder characterized by the repeated rise of concerns (obsessions) and repetitive unwanted behavior (compulsions). Although selective serotonin reuptake inhibitors (SSRIs) is the first-choice drug, response rates to SSRI treatment vary between symptom dimensions. In this study, to find a therapeutic target for SSRI-resilient OCD symptoms, we evaluated treatment responses of quinpirole (QNP) sensitization-induced OCD-related behaviors in mice. SSRI administration rescued the cognitive inflexibility, as well as hyperactivity in the lateral orbitofrontal cortex (lOFC), while no improvement was observed for the repetitive behavior. D_2_ receptor signaling in the central striatum (CS) was involved in SSRI-resistant repetitive behavior. An adenosine A_2A_ antagonist, istradefylline, which rescued abnormal excitatory synaptic function in the CS indirect pathway medium spiny neurons (MSNs) of sensitized mice, alleviated both of the QNP-induced abnormal behaviors with only short-term administration. These results provide a new insight into therapeutic strategies for SSRI-resistant OCD symptoms and indicate the potential of A_2A_ antagonists as a rapid-acting anti-OCD drug.

## Significance Statement

Clinical studies show distinct therapeutic efficacies for SSRIs between subtypes of obsessive-compulsive disorder (OCD) symptoms. While abnormal activity in the cortico-striatal pathway is critically involved in the pathophysiology of OCD, the neurologic mechanisms and therapeutic strategies for selective serotonin reuptake inhibitor (SSRI)-resistant symptoms remain unclear. In this study, we showed that repeated injection of dopamine D_2_ receptor agonist, quinpirole (QNP) elicited two distinct OCD-related behaviors: cognitive inflexibility (SSRI-responsive) and repetitive behavior (SSRI-resistant). While SSRI treatment normalized hyperactivity of the orbitofrontal cortex, we also demonstrated the imbalanced excitatory inputs in the central striatum (CS) of QNP-treated mice and the therapeutic potential of an A_2A_ antagonist as a modulator of indirect pathway medium spiny neurons (MSNs).

## Introduction

Obsessive-compulsive disorder (OCD) is a psychiatric disorder characterized by repetitive inappropriate thoughts (obsessions) and behaviors to get rid of obsessions (compulsions; Milad and Rauch, 2012; Bokor and Anderson, 2014). The lifetime prevalence of OCD is ∼2–3%, and most cases are childhood or adolescent onset (Milad and Rauch, 2012; Pauls et al., 2014). Although selective serotonin reuptake inhibitors (SSRIs) are the first-choice treatment for OCD, they require a longer time and higher dose before the onset of therapeutic effects for OCD treatment than for the treatment of major depression (Bokor and Anderson, 2014). Furthermore, even when SSRIs are used properly, 40–60% of patients are resistant to the therapy (Pallanti et al., 2004). Recent evidence has suggested that the efficacies of drug treatment vary by symptom dimensions. For instance, patients with aggression-related obsessions and checking compulsions respond well to SSRI treatment, while sexual/religious obsessions are associated with a poor treatment response (Starcevic and Brakoulias, 2008; Landeros-Weisenberger et al., 2010). In this situation, a novel anti-OCD drug is strongly desired but remains challenging.

OCD was originally classified as an anxiety disorder; however, GABA-enhancing anti-anxiety drugs are ineffective for OCD patients. Whereas anxiety symptoms in OCD patients are heterogeneous, recent clinical studies postulate that cognitive inflexibility might cause unstoppable obsessions and compulsions, highlighting distinct features of OCD and anxiety disorders (Van Ameringen et al., 2014). Based on these observations, OCD and related disorders have recently been recategorized as a stand-alone group characterized by repetitive behavior in the Diagnostic and Statistical Manual of Mental Disorder (DSM-V; Van Ameringen et al., 2014). Consistent with the updated diagnostic criteria, brain imaging studies have indicated that OCD patients show hyperactivity in cortico-striatal circuits, especially in the orbitofrontal cortex and caudate (Baxter et al., 1987; Graybiel and Rauch, 2000). Hyperactivity of the frontal cortex and striatum was only normalized in patients who responded to SSRI-treatment (Saxena et al., 1999); therefore, control of cortico-striatal pathway activity may be a key for understanding the pathophysiology of OCD and developing novel therapeutic targets for OCD.

Among existing experimental tools for the study of OCD, quinpirole (QNP)-induced psychosis in rats are known to be an easy-to-use tool (Stuchlik et al., 2016). After several injections of a dopamine D_2_ agonist, QNP, rats show several OCD-related behaviors, e.g., robust repetitive checking behavior, which is considered to be similar to the checking compulsion in OCD patients (Szechtman et al., 1998; Stuchlik et al., 2016). However, despite the good similarity in the behavioral phenotype, limited information regarding pharmacotherapeutic response, especially for SSRI treatment, is available (Stuchlik et al., 2016). Considering the limited efficacy of SSRI treatment against several OCD symptoms, an assessment of both SSRI-responsive and SSRI-resistant OCD-like behaviors is beneficial for the elucidation of the pathophysiological and therapeutic mechanisms of OCD.

In the present study, we applied the QNP sensitization protocols to mice and characterized OCD-related behavioral and neurologic abnormalities. QNP-treated mice showed OCD-like repetitive behavior, cognitive inflexibility, and hyperactivity of the pyramidal neurons in the lateral orbitofrontal cortex (lOFC). The cognitive inflexibility and lOFC hyperactivity were rescued by chronic, high-dose SSRI administration, whereas the repetitive behavior was not improved by SSRI administration. SSRI-resistant repetitive behavior was rescued by the local inhibition of D_2_ signaling in the central striatum (CS), a projection site of the lOFC. The short-term administration of an adenosine A_2A_ receptor antagonist, istradefylline rescued both of SSRI-responsive and SSRI-resistant OCD-like behaviors in QNP-treated mice. Finally, we showed that electrophysiological studies showed abnormal excitatory inputs to the CS in a cell type-specific manner and these abnormalities were improved by A_2A_ receptor antagonism. The present results offer a new insight into the therapeutic strategy for treatment-resistant OCD.

## Materials and Methods

### Reagents

DL-2-amino-5-phosphonopentanoic acid (DL-APV; an NMDA antagonist; Sigma-Aldrich) and tetrodotoxin (a voltage-dependent Na^+^ channel blocker; Sigma-Aldrich) were dissolved in water. (-)-QNP (a dopamine D_2_ agonist; Tocris Bioscience) was dissolved in water (for *ex vivo* recordings) or saline (for intraperitoneal injection). 6,7-Dinitroquinoxaline-2,3(1H,4H)-dione (DNQX; an AMPA antagonist; Tocris Bioscience), bicuculline (a GABA_A_ antagonist; Enzo Life Science), raclopride (a D_2_ antagonist; Abcam Biochemicals), PD98059 [a mitogen-activated protein kinase kinase (MEK) inhibitor; Cayman Chemical Company], and CGS 21680A (an A_2A_ receptor agonist; Toronto Research Chemicals) were dissolved in dimethyl sulfoxide (DMSO). Stock solutions were stored at –20°C until use and dissolved in saline, artificial CSF (ACSF), or pipette solution. The final concentration of DMSO was lower than 5% for intraperitoneal injection and microinjection and 0.05% for electrophysiology.

### Animals

All animal care and experimental procedures were conducted in accordance with the ethical guidelines of the Kyoto University Animal Research Committee. Male C57BL/6JJmsSlc mice which are the C57BL/6J substrain mice (RRID:IMSR_JAX:000664) maintained at Nihon SLC were purchased and housed at a constant ambient temperature of 24 ± 1°C on a 12/12 h light/dark cycle with access to food and water *ad libitum*. For behavioral experiments, mice over seven weeks old were used. For the spatial discrimination task, habituation was started at five weeks old or older, and training was started at seven weeks old or older. For electrophysiological recordings, 7- to 12-week-old mice were used.

For QNP sensitization, mice were intraperitoneally injected with QNP (1 mg/kg) every weekday. For rat, a dose of 0.5 mg/kg was usually used (Szechtman et al., 1998; Servaes et al., 2017). We calculated a dose for mice based on the body surface area (Nair and Jacob, 2016). Mice that received more than eight injections of QNP were considered QNP-sensitized mice.

For chronic antidepressant treatment, citalopram hydrobromide (FWD Chemicals) was dissolved in drinking water (0.2 mg/ml) and administered for 28 d, resulting in an average dose of 24 mg/kg/d (Asaoka et al., 2017). The drug-containing drinking water was shielded from light and changed every 3–5 d.

For the short-term administration of diazepam (0.3 mg/kg), citalopram (10 mg/kg), and istradefylline (3 mg/kg), the drug was intraperitoneally injected 5 min before QNP injection.

### Recording of repetitive behavior

Mice were singly or pair-housed, and spontaneous behavior in their home cage was videotaped. Chewing the cage bedding (wood chip) or, in rare cases, the cage mate’s hair was considered repetitive (ritual) behavior. Repeated chewing behavior consisted of the following behaviors; holding a wood chip (or fur) in the forelimbs and gently biting and pulling the chip (or hair) by the mouth and forelimbs. At first, we chose the pair-housed condition to reduce stress, but aggressive behavior toward the cage mate was sometimes observed (in both vehicle-treated and drug-treated groups). Therefore, in later experiments, mice were singly housed. There was no apparent difference in repetitive behavior between pair-housed and singly housed mice (pair-housed mice: 516.8 ± 15.1 s, *n* = 16; singly housed mice: 532.4 ± 18.38, *n* = 19, *p* = 0.5256 by Student’s *t* test).

### Spatial discrimination learning and reversal learning

For the spatial discrimination task, mice were food restricted (2–3 g/d) on weekdays (80–90% of the *ad libitum* body weight; Miyazaki et al., 2014). On the weekend, food was freely available.

For the habituation of the mice to the reward (sweetened milk), mice were allowed free access to sweetened milk for ∼30–60 min. After the 2-d habituation to the reward, mice received pre-training for 4–6 d. In the pre-training period, mice were placed in the T-maze, which consisted of one start arm (30 × 10 cm), two goal arms (30 × 10 cm), and 30-cm-high surrounding walls, and were allowed to freely explore. Both goal arms were rewarded during the pre-training period.

Spatial discrimination tests were performed as previously described (Moy et al., 2007; Bannerman et al., 2008) with several modifications. Mice received 6 or 7 d training and 8 d overtraining (Smith et al., 2012). During these periods, mice were trained for five free-choice trials per day. The rewarded goal arm (rewarded with 100 μl of sweetened milk) was randomly chosen and fixed during the training and overtraining periods. At the entrance of each goal arm, a guillotine door was placed, and once the mice entered the goal arm, the door was immediately closed. Mice were returned to their home cage during the preparation for the next trial (∼2 min).

On the 7th or 8th training day, the correct choice rate during the previous 3 d was calculated, and mice that showed a correct choice rate of >75% were used for the subsequent overtraining. The day that the mice met this criterion was considered to be day 1 of the overtraining period (OT1).

During the overtraining period, mice received similar spatial discrimination training as in the previous training period combined with QNP injection (1 mg/kg, i.p.). The effects of reduced locomotion by an acute QNP injection were avoided by injecting QNP after training on the first two to three overtraining days (OT1–OT2 or OT3) and then 20–30 min after training on OT3 or OT4–OT8. For the second criterion, the correct choice rate during OT4–OT8 was calculated, and mice that showed a correct choice rate of >80% were used for the reversal learning test.

For reversal learning, the rewarded arm was reversed, and mice underwent 10 free-choice trials per day for 4 d (R1–R4). During this period, QNP was injected 20–30 min before starting experiments.

The spatial discrimination task without an overtraining period consisted of an 8-d training period (T1–T8) and a 4-d reversal learning period (R1–R4). QNP was injected after (T1–T2) and before (T3–T8, R1–R4) experiments.

### Elevated plus maze test

The elevated plus maze consisted of two open arms and two closed arms (30 × 5 cm) extended from a central platform (5 × 5 cm). After 25 min of drug injection, mice were placed on the central platform and monitored for 5 min. The time spent in each arm was analyzed using a video tracking system (ANY-maze version 4.99).

### Open field test

After 25 min of drug injection, mice were placed at the center of an open field (75 × 75 cm; without a wall; Szechtman et al., 1994) and monitored for 10 min. The total distance traveled was analyzed using a video tracking system (ANY-maze version 4.99).

### Preparation of the adeno-associated virus (AAV) vector

Lenti-X 293T cells were transfected with pAAV-hSyn1-Venus, pAAV-DJ, and pHelper using polyethylenimine (polyethylenimine “Max,” Polysciences), and 72 h after transfection, the cells were gently scraped with a gradient buffer (1 mM Tris, 15 mM NaCl, and 1 mM MgCl_2_). The buffer was freeze-thawed four times between liquid nitrogen and a 55°C water bath to break the cell membrane. DNA and RNA were removed by benzonase nuclease (Sigma), and cell debris was removed by centrifugation at 3000 × *g* for 15 min. Viral stocks were purified using four different layers of an iodixanol (Opti Prep, Sigma) gradient, i.e., 15%. 25%, 40%, and 58%. After ultracentrifugation for 105 min at 48,000 rpm, the viral fraction was extracted from the interface between the 40% and 58% layers.

### Stereotaxic surgery and microinjection

Mice were anesthetized with sodium pentobarbital (50 mg/kg, i.p., Nakarai Tesque) and fixed on a small animal stereotaxic frame (Narishige). For lOFC neuronal labeling, 0.75-μl AAV-hSyn1-Venus was microinjected into the lOFC (AP +2.7 mm, ML +1.7 mm, DV +2.7 mm from bregma). After four weeks, mice were decapitated, and coronal forebrain slices were prepared by using a vibratome (see below, Preparation of acute brain slices for electrophysiological analysis). Forebrain slices were fixed in 4% paraformaldehyde. After fixation, slices were washed in phosphate-buffered saline, and the green fluorescence of Venus was visualized using a Nikon Diaphot 200 microscope equipped with a laser scanning confocal imaging system (MRC-1024, Bio-Rad Laboratories).

For drug microinjection, mice were implanted with a bilateral guide cannula directed at the central striatum (CS; AP +1.2 mm, ML +2.0 mm, DV +3.8 mm from bregma, angled 10°) and fixed to the skull by dental cement. On the experimental day, the injection cannula was inserted into the guide, and drug (1-μg raclopride or PD98059 in 1 μl or 0.3-ng CGS 21680A in 1 μl) was injected at a rate of 0.15 μl/min. After injection, the injection cannula was left in place for for 5 min (for raclopride) or 10 min (for PD98059). For CGS 21680A, the injection cannula was left during the recording. After experiments, 0.5 μl of Evans Blue solution was injected through the cannula to confirm the injection site. When injection site was incorrect, the animal was excluded from analysis.

### Preparation of acute brain slices for electrophysiological analysis

For electrophysiological analysis, mice were received eight injections of QNP or saline and the next day after the 8th injection, acute brain slices were prepared. Mice were deeply anesthetized with isoflurane and decapitated. The brains were rapidly collected in ice-cold cutting solution (120 mM N-methyl-D-glucamin-Cl, 2.5 mM KCl, 26 mM NaHCO_3_, 1.25 mM NaH_2_PO_4_, 0.5 mM CaCl_2_, 7 mM MgCl_2_, 15 mM D-glucose, and 1.3 mM ascorbic acid; pH 7.2). Coronal brain slices (200-μm thick) were prepared with a vibratome (VT1000S, Leica). For recording from the CS, slices were dissected from relatively anterior part of the striatum, where OFC send dense projections (Hunnicutt et al., 2016). Slices were recovered in oxygenated ACSF (124 mM NaCl, 3 mM KCl, 26 mM NaHCO_3_, 1 mM NaH_2_PO_4_, 2.4 mM CaCl_2_, 1.2 mM MgCl_2_, and 10 mM D-glucose; pH 7.3) at 32°C for at least 1 h before recording. After recovery, individual slices were transferred to a recording chamber with continuous perfusion of oxygenated ACSF at a flow rate of 1–2 ml/min. ACSF was warmed to keep the recording chamber at 27 ± 1°C. Recordings were performed only within 4 h after recovery.

### Electrophysiological recordings

Electrophysiological recordings were performed with an EPC9 amplifier (HEKA), and the data were recorded using Patchmaster software (HEKA). The resistance of the electrodes was 3–6 MΩ when filled with the internal solution (140 mM K-gluconate, 5 mM KCl, 10 HEPES, 2 mM Na-ATP, 2 mM MgCl_2_, and 0.2 mM EGTA; pH 7.3 adjusted with KOH for current-clamp recordings and EPSC recordings from the lOFC; 70 mM K-gluconate, 75 mM KCl, 10 mM HEPES, 2 mM Na-ATP, 2 mM MgCl_2_, and 0.2 mM EGTA; pH 7.3 adjusted with KOH for IPSC recordings; and 120 mM CsMeSO_4_, 15 mM CsCl, 8 mM NaCl, 10 mM HEPES, 2 mM Mg-ATP, 0.3 mM Na-GTP, 0.2 mM EGTA, 10 mM TEA-Cl, and 5 mM QX-314; pH 7.3 adjusted with CsOH for EPSC recordings from the striatum). Individual neurons were visualized with a microscope equipped with a 40× water-immersion objective lens (Carl Zeiss) and a CCD camera. The series resistance was compensated by 70% and maintained within 35 MΩ.

**Figure 1.**
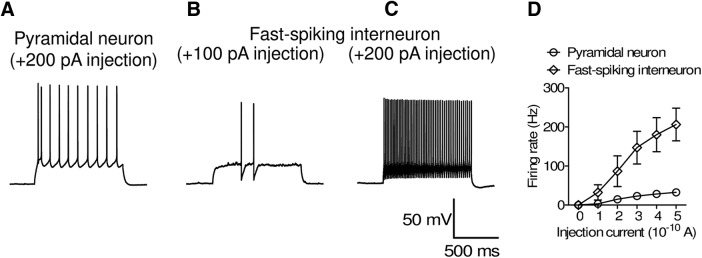
Electrophysiological characteristics of lOFC pyramidal neurons and fast-spiking interneurons (***A–C***) Representative firing activity recorded from a pyramidal neuron (***A***; 200-pA injection) and fast-spiking interneuron (***B***; 100-pA injection, ***C***; 200-pA injection). ***D***, Current injection-induced firing activity of pyramidal neurons and fast-spiking interneurons. Please note that the data set for pyramidal neurons is same as that in Figure 4*B* (saline group). (Pyramidal neurons; *n* = 10 from 3 mice, fast-spiking interneurons; *n* = 5 from 3 mice.)

For recording from lOFC pyramidal neurons, a current injection (100–300 pA, 1-s duration) was performed to elicit action potentials. As previously reported (Tateno and Robinson, 2006), pyramidal neurons showed regular-spiking activity (Fig. 1*A*,*D*), whereas interneurons showed fast-spiking activity (Fig. 1*B–D*). lOFC neurons showing regular-spiking activity were used for experiments. Action potentials were evoked by current injection (0–500 pA, 1-s duration). EPSCs were recorded with bath application of the GABA_A_ antagonist (20 μM bicuculline), while AMPA/NMDA antagonists (20 μM DNQX and 50 μM APV) were applied to record IPSCs. Tetrodotoxin (0.3 μM) was added to the bath solution for recording miniature EPSCs and IPSCs. Events were analyzed by Minianalysis software (SynaptoSoft). The membrane potential during voltage-clamp recordings was held at –70 mV.

For the recordings from CS medium spiny neurons (MSNs), MSNs were determined by their morphologic features, and after the recording, single-cell PCR was performed to identify the cell type. For acute QNP treatment, QNP (10 μM) was bath applied for at least 3 min. For electrical stimulation, a stimulation electrode was placed near the recording electrode. AMPA-mediated eEPSCs and mixed AMPA and NMDA-mediated eEPSCs were evoked by stimulation at –70 and +40 mV, respectively. NMDA-mediated eEPSC amplitude was determined as the average amplitude between 45 and 55 ms after stimulation. The average of three NMDA/AMPA ratio measurements was used for analysis.

### Single-cell reverse transcription-polymerase chain reaction (RT-PCR)

**Figure 2.**
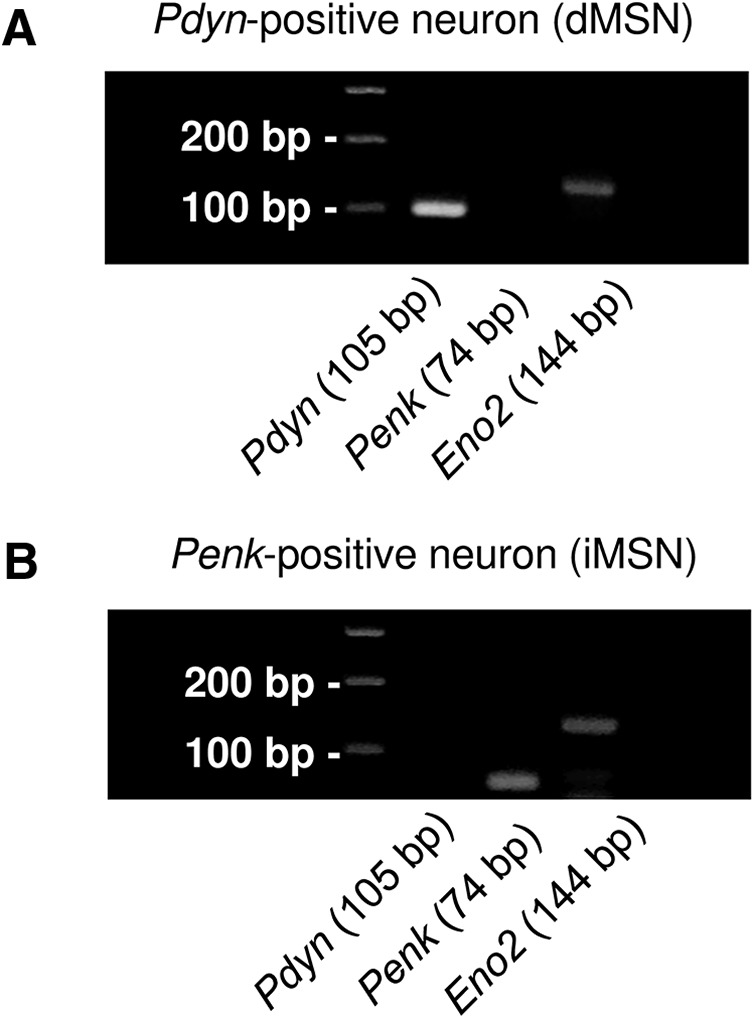
Representative single-cell PCR from a CS dMSN and iMSN. Representative image of single-cell PCR from CS MSNs. *Pdyn*-positive neurons were considered direct-pathway MSNs (dMSNs; ***A***), while *Pdyn*-negative and *Penk*-positive neurons were considered indirect-pathway MSNs (iMSNs; ***B***).

After the whole-cell recording, the contents of the cell were aspirated into the recording pipette and harvested in a sampling tube. The collected samples were reverse-transcribed using a ReverTra Ace RT kit (TOYOBO) and amplified with Blend Taq (TOYOBO). The oligonucleotide primers used were 5'- CCCAGGCGACATCAATTT-3' and 5'-TCTCCCAGATTTTGAAAGAAGG-3' for proenkephalin (*Penk*); 5'-CCAGGGACAAAGCAGTAAGC-3' and 5'-CGCCATTCTGACTCACTTGTT-3' for prodynorphin (*Pdyn*); and 5'-CCGCTGATCCTTCCCGATAC-3' and 5'-CGACGTTGGCTGTGAACTTG-3' for enolase 2 (*Eno_2_*) as a neuronal marker. The PCR products were analyzed using agarose gel electrophoresis. *Pdyn*-positive neurons were considered to be direct pathway MSNs (dMSNs), and *Pdyn*-negative and *Penk*-positive neurons were considered to be indirect pathway MSNs (iMSNs; Fig. 2*A*,*B*).

### Experimental design and statistical analysis

All data are presented as the mean ± SEM. Statistical analysis was performed with GraphPad Prism 5 (GraphPad; RRID:SCR_002798). Differences with *p* < 0.05 were considered significant. The differences between two groups were compared by a two-tailed Student’s *t* test or unpaired *t* test with Welch’s correction. When differences within a mouse were compared, a two-tailed paired *t* test was used for analysis. The differences between more than three groups were compared by one-way ANOVA with *post hoc* Tukey’s multiple comparison test. For examination of the time course or current injection experiments, two-way ANOVA for repeated measures and following Bonferroni *post hoc* test was used for analysis. Before performing repeated measures ANOVA, Mauchly’s sphericity test were performed by using R (version 3. 5. 2; RRID:SCR_001905) and when the assumption of sphericity is violated, the Greenhouse–Geisser correction was used. Changes in the NMDA/AMPA ratio were analyzed by one-sample *t* test.

## Results

### Repeated injection of QNP induced OCD-related behaviors and lOFC hyperactivity

**Figure 3.**
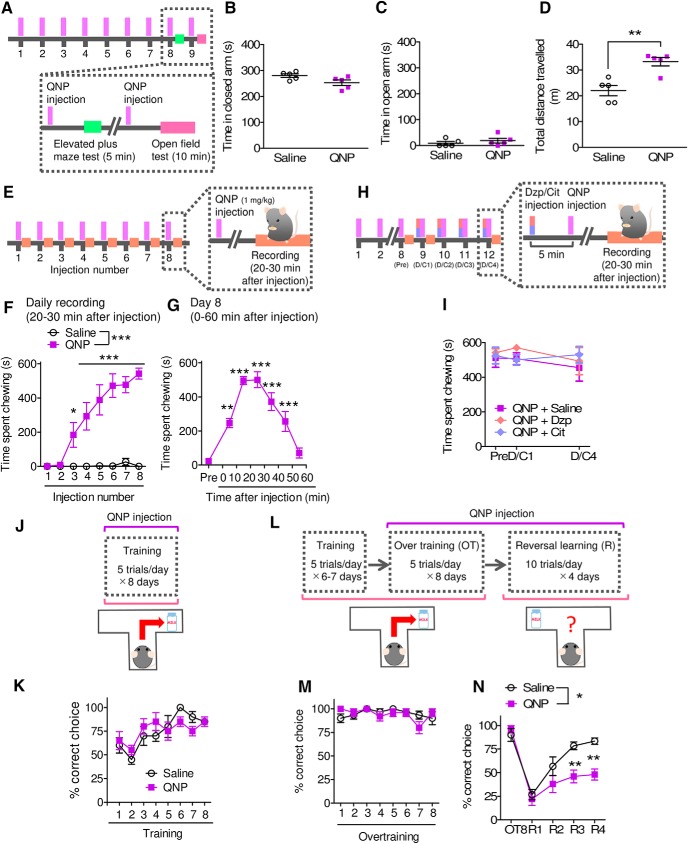
Repeated injection of QNP elicited multiple OCD-related symptoms. ***A***, Time course of the elevated plus maze test and open field test. ***B***, ***C***, Time spent in the closed arm (***B***) and the open arm (***C***) in an elevated plus maze test. ***D***, Total travel distance in the open field test. (Saline; *n* = 5, QNP; *n* = 5, ***B***: Student’s *t* test; *t*_(8)_ = 2.178, *p* = 0.0610, ***C***: Student’s *t* test; *t*_(8)_ = 0.8863, *p* = 0.4013, ***D***: Student’s *t* test; *t*_(8)_ = 4.343, **p = 0.0025.). ***E***, Time course of recording of QNP-induced repetitive behavior. ***F***, ***G***, Time spent chewing during the 20–30 min after the 1st–8th QNP injection (***F***) and before and after the 8th QNP injection (***G***). [***F***: saline; *n* = 6, QNP; *n* = 7, two-way repeated measures ANOVA; drug (*F*_(1,24.32)_ = 37.18, *p* < 0.0001), injection number (*F*_(2.21,53.75)_ = 22.41, *p* < 0.0001), interaction (*F*_(2.21,53.75)_ = 20.95, *p* < 0.0001), Bonferroni *post hoc* test; *p < 0.05 and ***p < 0.001, ***G***: *n* = 4, one-way repeated measures ANOVA; *F*_(6,18)_ = 38.61, *p* < 0.0001, Tukey’s multiple comparison test; **p < 0.01, **p < 0.001 vs Pre.] ***H***, Time course of recording for QNP-induced repetitive behavior combined with short-term administration of diazepam and citalopram. ***I***, Effects of the short-term administration of an antianxiety agent, diazepam (Dzp; 0.3 mg/kg) and an antidepressant, citalopram (Cit; 10 mg/kg) on repetitive behavior in QNP-treated mice. [QNP+saline; *n* = 4, QNP+Dzp; *n* = 4, QNP+Cit; *n* = 4, two-way repeated measures ANOVA; drug (*F*_(2,36)_ = 0.41, *p* = 0.6748), injection number (*F*_(2,36)_ = 0.56, *p* = 0.5786), interaction (*F*_(2,36)_ = 0.42, *p* = 0.7918).] ***J***, ***K***, Protocols and percentage of correct choice during training with daily injection of QNP. (Saline; *n* = 4, QNP; *n* = 4, ***K***: two-way repeated measures ANOVA; drug (*F*_(1,20.23)_ = 0.01, *p* = 0.9224), injection number (*F*_(3.37,68.17)_ = 9.04, *p* = 0.0004), interaction (*F*_(3.37,68.17)_ = 1.66, *p* = 0.2037).] ***L***, Protocols of a spatial discrimination task and a reversal learning test. ***M***, ***N***, Percentage of correct choice during overtraining (***M***) and reversal learning (***N***). [Saline; *n* = 6, QNP; *n* = 5, ***M***: two-way repeated measures ANOVA; drug (*F*_(1,25.63)_ = 0.07, *p* = 0.8025), session number (*F*_(2.85,73.04)_ = 1.93, *p* = 0.1519), interaction (*F*_(2.85,73.04)_ = 1.58, *p* = 0.2201), ***N***: two-way repeated measures ANOVA; drug (*F*_(1,36)_ = 10.46, *p* = 0.0102), session number (*F*_(4,144)_ = 34.86, *p* < 0.0001), interaction (*F*_(4,144)_ = 4.29, *p* = 0.0061), Bonferroni *post hoc* test; **p < 0.01.]

First, we produced QNP-sensitized mice and characterized their behavioral and neurologic changes. Mice received a QNP (1 mg/kg) injection every weekday, and after eight to nine injections, QNP-treated mice showed more locomotor activity in the open field than saline-treated mice (Fig. 3*A*,*D*) but did not display any anxiogenic effects in the elevated-plus maze test (Fig. 3*A–C*). Hyper-locomotion in the open field is reported to be a feature of QNP sensitization in rats (Szechtman et al., 1994), and thus, this result was indicative of the successful establishment of QNP sensitization in mice.

Repetitive behaviors are one of the widely accepted OCD-related behaviors in rodents (Boulougouris et al., 2009; Camilla d'Angelo et al., 2014; Zike et al., 2017). Following three to four injections of QNP, mice showed repeated chewing behavior in their home cages (chewing wood chip bedding or cage mate’s hair; see Materials and Methods, Recording of repetitive behavior). This repetitive behavior peaked after eight injections (Fig. 3*E*,*F*) . Additional injection of QNP (total 9–12 injections) did not induce further changes in the duration of chewing behavior (Fig. 3*I*, QNP+saline group). This robust chewing was only observed after the QNP injection and was eliminated within 60 min of the injection (Fig. 3*G*). Therefore, the repeated injection and challenge with QNP induced repetitive behavior. In addition, the short-term administration of diazepam (0.3 mg/kg) and citalopram (10 mg/kg), which do not show therapeutic effects in OCD patients, had no effect on the chewing behavior (Fig. 3*H*,*I*).

Recent clinical evidence has suggested that OCD patients exhibit cognitive inflexibility and increased reliance on habitual responses (Gillan et al., 2011, 2016). To assess this feature, we performed a spatial discrimination and reversal learning task. Daily QNP injection did not affect spatial learning (Fig. 3*J*,*K*), indicating that the repeated QNP injection did not affect goal-directed learning. In a spatial discrimination task, longer training period enhances habitual learning (Smith et al., 2012). To assess whether QNP-treated mice showed cognitive inflexibility after longer learning period, mice received modest overtraining (five trials per day, 8 d) after the training period (Fig. 3*L*,*M*). Under this condition, saline-treated mice still showed flexible behavior, while QNP-treated mice displayed a deficit in reversal learning (Fig. 3*N*), indicating that QNP-treated mice easily exhibit habit-like inflexible behavior.

**Figure 4.**
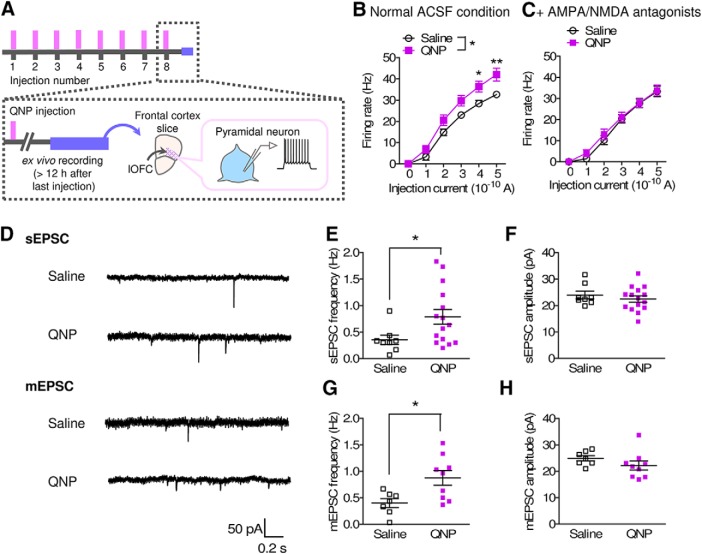
Hyperactivity of lOFC pyramidal neurons in QNP-treated mice. ***A***, Time course of electrophysiological recordings. ***B***, ***C***, Current injection induced firing activity of lOFC pyramidal neurons in the absence (***B***) and presence (***C***) of AMPA/NMDA antagonists. [***B***: saline; *n* = 10 from 3 mice, QNP; *n* = 10 from 3 mice, two-way repeated measures ANOVA; drug (*F*_(1,36.27)_ = 6.15, *p* = 0.0227), current (*F*_(1.91,69.27)_ = 324.00, *p* < 0.0001), interaction (*F*_(1.91,69.27)_ = 4.07, *p* = 0.0270), Bonferroni *post hoc* test; *p < 0.05 and **p < 0.01, ***C***: saline; *n* = 11 from 3 mice, QNP; *n* = 10 from 3 mice, two-way repeated measures ANOVA; drug (*F*_(1,38.97)_ = 0.03, *p* = 0.8743), current (*F*_(2.29,89.24)_ = 209.69, *p* < 0.0001), interaction (*F*_(2.29,89.24)_ = 0.45, *p* = 0.6660).] ***D***, Representative traces of spontaneous EPSCs (sEPSCs) and miniature EPSCs (mEPSCs). ***E***, ***F***, sEPSC frequency (***E***) and amplitude (***F***) in lOFC pyramidal neurons. (Saline; *n* = 8 from 3 mice, QNP; *n* = 15 from 3 mice, ***E***: unpaired *t* test with Welch’s correction; *t*_(21)_ = 2.632, *p = 0.0160, ***F***: Student’s *t* test; *t*_(21)_ = 0.7675, *p* = 0.4513.) ***G***, ***H***, mEPSC frequency (***G***) and amplitude (***H***) in lOFC pyramidal neurons. (Saline; *n* = 7 from 3 mice, QNP; *n* = 9 from 3 mice, ***G***: Student’s *t* test; *t*_(14)_ = 2.740, *p = 0.0160, ***H***: Student’s *t* test; *t*_(14)_ = 1.277, *p* = 0.2223.).

Clinical studies have suggested that activity in the lOFC is higher in OCD patients than in healthy controls and that successful SSRI treatment normalizes this activity (Baxter et al., 1987; Saxena et al., 1999). To determine whether QNP-treated mice show OCD-like neurologic abnormalities, the firing activity was recorded by using *ex vivo* electrophysiological recording from lOFC pyramidal neurons (Fig. 4*A*). lOFC pyramidal neurons from QNP-treated mice showed a higher firing response than those from saline-treated mice. This increase was abolished in the presence of AMPA and NMDA antagonists (20 μM DNQX and 50 μM AP-V; Fig. 4*B*,*C*). There was no difference between the resting membrane potential of pyramidal neurons from QNP and saline-treated mice (saline group; –80.41 ± 1.23 mV, *n* = 10 from 3 mice, QNP group; –81.25 ± 2.02 mV, *n* = 11 from 3 mice, *p* = 0.7358 by Student’s *t* test). Both the spontaneous and miniature EPSC frequencies in lOFC pyramidal neurons were significantly higher in QNP-treated mice than in saline-treated mice (Fig. 4*D*,*E*,*G*), while no change in the EPSC amplitude was observed (Fig. 4*F*,*H*), suggesting a plastic change in the glutamatergic synapses in the lOFC of QNP-treated mice.

### Chronic SSRI administration rescued the cognitive inflexibility and neurologic deficits but not the repetitive behavior in QNP-treated mice

**Figure 5.**
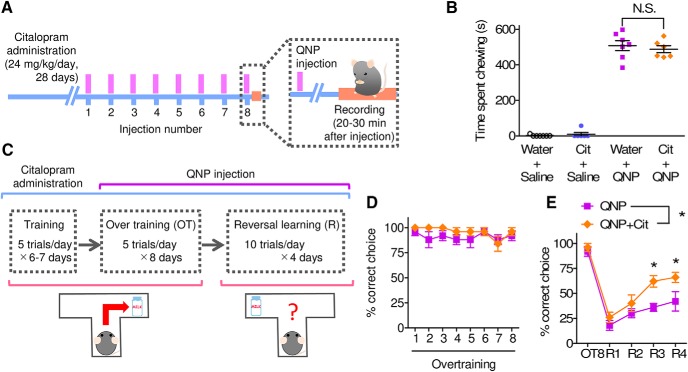
Chronic SSRI treatment rescued cognitive inflexibility in QNP-treated mice but not the abnormal repetitive behavior. ***A***, Time course of recording for QNP-induced repetitive behavior combined with chronic SSRI administration. ***B***, Time spent chewing during the 20–30 min after the 8th QNP injection. [Water+saline; *n* = 7, Cit+saline; *n* = 6, water+QNP; *n* = 7, Cit+QNP; *n* = 6, two-way ANOVA; p.o. administration (*F*_(1,22)_ = 0.56, *p* = 0.4616), i.p. injection (*F*_(1,22)_ = 727.10, *p* < 0.0001), interaction (*F*_(1,22)_ = 0.11, *p* = 0.7440), Bonferroni *post hoc* test; not significant (n.s.).] ***C***, Time course of a spatial discrimination task and a reversal learning test combined with chronic SSRI administration. ***D***, ***E***, Percentage of correct choices during overtraining (***D***) and reversal learning (***E***). [Water+QNP; *n* = 5, Cit+QNP; *n* = 5, ***D***: two-way repeated measures ANOVA; drug (*F*_(1,26.9)_ = 2.47, *p* = 0.1547), session number (*F*_(3.36,90.38)_ = 1.27, *p* = 0.3040), interaction (*F*_(3.36,90.38)_ = 0.62, *p* = 0.6233), ***E***: two-way repeated measures ANOVA; drug (*F*_(1,32)_ = 9.71, *p* = 0.0143), session number (*F*_(4,128)_ = 48.66, *p* < 0.0001), interaction (*F*_(4,128)_ = 1.63, *p* = 0.1920), Bonferroni *post hoc* test; *p < 0.05.).]

To examine the treatment response to a high dose of an SSRI, mice were treated with citalopram (24 mg/kg/d) for 28 d. In QNP-treated mice, although the SSRI failed to reduce the repetitive chewing behavior (Fig. 5*A*,*B*), SSRI treatment improved the reversal learning in the spatial discrimination task combined with overtraining (Fig. 5*C–E*).

**Figure 6.**
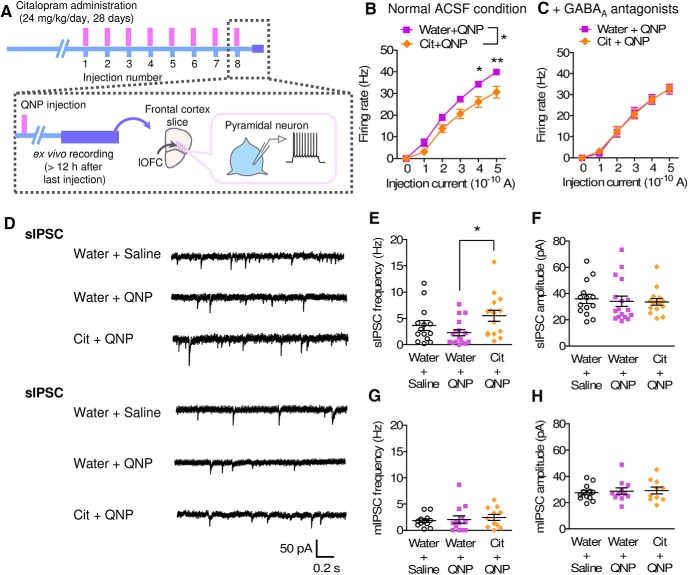
Chronic SSRI treatment rescued lOFC hyperactivity in QNP-treated mice. ***A***, Time course of electrophysiological recordings combined with chronic SSRI administration. ***B***, ***C***, Current injection induced firing activity of lOFC pyramidal neurons in the absence (***B***) and presence (***C***) of GABA_A_ antagonists. [***B***: water+QNP; *n* = 8 from 3 mice, Cit+QNP; *n* = 12 from 3 mice, two-way repeated measures ANOVA; drug (*F*_(1,27.96)_ = 6.96, *p* = 0.0167), current (*F*_(1.55,43.34)_ = 309.92, *p* < 0.0001), interaction (*F*_(1.55,43.34)_ = 4.04, *p* = 0.0297), Bonferroni *post hoc* test; *p < 0.05 and **p < 0.01, ***C***: water+QNP; *n* = 11 from 3 mice, Cit+QNP; *n* = 11 from 3 mice, two-way repeated measures ANOVA; drug (*F*_(1,40.58)_ = 0.00, *p* = 0.9673), current (*F*_(2.03,82.38)_ = 250.93, *p* < 0.0001), interaction (*F*_(2.03,82.38)_ = 0.12, *p* = 0.8889).] ***D***, Representative traces of sIPSCs and mIPSCs. ***E***, ***F***, sIPSC frequency (***E***) and amplitude (***F***) in lOFC pyramidal neurons. (Water+saline; *n* = 14 from 3 mice, water+QNP; *n* = 17 from 3 mice, Cit+QNP; *n* = 15 from 3 mice, ***E***: one-way ANOVA; *F*_(2,43)_ = 3.758, *p = 0.0313, Tukey’s multiple comparison test; *p < 0.05, ***F***: one-way ANOVA; *F*_(2,43)_ = 0.1188, *p* = 0.8883.) ***G***, ***H***, mIPSC frequency (***G***) and amplitude (***H***) in lOFC pyramidal neurons. (***G***: water+saline; *n* = 12 from 3 mice, water+QNP; *n* = 13 from 3 mice, Cit+QNP; *n* = 11 from 3 mice, one-way ANOVA; *F*_(2,33)_ = 0.2934, *p* = 0.7476, ***H***: water+saline; *n* = 12 from 3 mice, water+QNP; *n* = 11 from 3 mice, Cit+QNP; *n* = 10 from 3 mice, one-way ANOVA; *F*_(2,30)_ = 0.1310, *p* = 0.8777.).

In electrophysiological recordings, SSRI treatment decreased the firing activity of lOFC pyramidal neurons in QNP-treated mice (Fig. 6*A*,*B*). The inhibitory effect of SSRI treatment was suppressed by a GABA_A_ antagonist (Fig. 6*C*). No differences were observed in the spontaneous IPSC amplitude or in the miniature IPSC frequency and amplitude between SSRI-treated and treatment-free QNP-treated mice (Fig. 6*D*,*F–H*), whereas the spontaneous IPSC frequency was increased in the SSRI plus QNP-treated mice compared to that in the QNP-only treated mice (Fig. 6*E*), suggesting that SSRI treatment increased the GABAergic inhibition of lOFC pyramidal neurons.

### D_2_-ERK signaling in the CS is involved in SSRI-resistant repetitive behavior in QNP-treated mice

**Figure 7.**
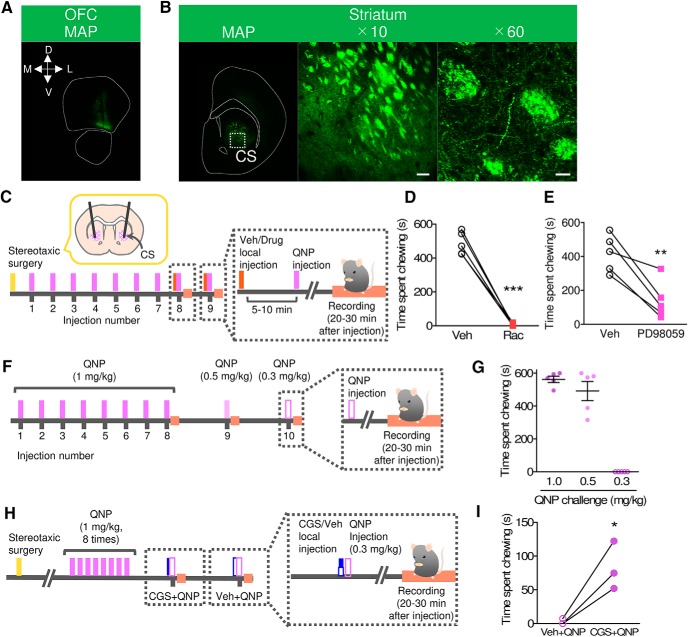
D_2_-ERK signaling in the CS was required for repetitive behavior in QNP-treated mice. ***A***, ***B***, Representative images from AAV-hSyn-Venus mediated labeling of lOFC neurons. Green fluorescence was observed at both the AAV injection site (***A***; lOFC) and the striatal projection site (***B***; CS). Scale bar = 100 μm (***B***, center) and 20 μm (***B***, right). ***C***, Time course of stereotaxic surgery and recording of QNP-induced repetitive behavior combined with intra-CS local drug injection. ***D***, ***E***, Effect of intra-CS injection of racroprode (Rac; 1 μg/side) or PD98059 (1 μg/side) on repetitive chewing behavior in QNP-treated mice. (***D***: *n* = 5, paired *t* test; *t*_(5)_ = 15.31, ***p < 0.0001, ***E***: *n* = 5, paired *t* test; *t*_(5)_ = 3.643, **p = 0.0070.) ***F***, Time course of recording of low-dose QNP-induced chewing behavior. ***G***, Time spent chewing during the 20–30 min after the 8th–10th QNP injection (1.0, 0.5, and 0.3 mg/kg, respectively); *n* = 5. ***H***, Time course of stereotaxic surgery and recording of intra-CS local injection-induced repetitive behavior combined with subthreshold dose of QNP injection. ***I***, Time spent chewing during the 20–30 min after intra-CS injection of CGS 21680A (CGS; 0.3 ng/side) and subthreshold dose of QNP injection (0.3 mg/kg); *n* = 3, paired *t* test; *t*_(2)_ = 4.395, *p = 0.0481.

Although lOFC hyperactivity was improved by chronic SSRI treatment, the repetitive chewing behavior was not reversed. Since the OFC-striatum pathway is activated in OCD patients (Baxter et al., 1987; Beucke et al., 2013), we hypothesized that synaptic changes in the lOFC-striatum pathway might be involved in the chewing behavior in QNP-treated mice. To test this hypothesis, we first confirmed the projection site of lOFC neurons in the striatum. For lOFC neuronal labeling, an AAV that expressed Venus protein (AAV-hSyn-Venus) was injected into the lOFC (Fig. 7*A*). Consistent with a previous report (Gremel et al., 2016), green fluorescence-positive terminals were observed in the central part of the striatum (CS), which is functionally classifies as a part of the associative striatum (Chuhma et al., 2017), indicating the presence of lOFC inputs in the CS (Fig. 7*B*).

Robust chewing behavior was observed only after challenge with QNP, suggesting that not only chronic changes but also the stimulation of D_2_ receptors were necessary for the induction of chewing behavior. To identify the contribution of D_2_ receptors in the CS, we performed a local bilateral injection of a D_2_ antagonist, raclopride (1 μg/side), in the CS (Fig. 7*C*). After the raclopride injection, QNP failed to elicit repetitive chewing in QNP-treated mice (Fig. 7*D*), indicating that D_2_ receptor signaling in the CS is required for repetitive chewing in QNP-treated mice.

In striatal neurons, the stimulation of D_2_ receptors activates extracellular signal-regulated kinase (ERK; Brami-Cherrier et al., 2002; Shioda et al., 2017). The local bilateral injection of a MEK/ERK inhibitor, PD98059 (1 μg/side), in the CS significantly reduced QNP-induced chewing behavior (Fig. 7*E*), suggesting the involvement of D_2_ receptor signaling-induced activation of the MEK/ERK pathway in repetitive chewing.

Adenosine A_2A_ receptors are G_s_-coupled receptors that modulate ERK activation (Moreau and Huber, 1999). Because, in the striatum, A_2A_ receptors are expressed in iMSNs but not dMSNs (Calabresi et al., 2014), we hypothesized that stimulation of A_2A_ receptors facilitates D_2_ receptor stimulation-induced chewing behavior in QNP sensitized mice. To test this hypothesis, we examined the effects of intra-CS injection of an A_2A_ receptor agonist on subthreshold dose of QNP-induced behavior. For the definition of the subthreshold dose of QNP, we examined three different doses of QNP (1.0, 0.5, 0.3 mg/kg, i.p.). After seven-time injection of normal dose of QNP (1 mg/kg), mice were received different dose of QNP challenge (Fig. 7*F*). At the dose of 0.5 mg/kg, there seemed a slight decrease in chewing behavior, whereas chewing behavior was not observed at 0.3 mg/kg (Fig. 7*G*). From these results, we defined a dose of 0.3 mg/kg as subthreshold dose of QNP. Next, we examined the effects of a combination of an A_2A_ receptor agonist (CGS 21680A; CGS) and subthreshold dose of QNP. After sensitization, CGS (0.3 ng/side) or vehicle was infused into the CS and concurrently, subthreshold dose of QNP (0.3 mg/kg, i.p.) was injected (Fig. 7*H*). CGS + QNP elicit significant increase in chewing behavior compared to Veh + QNP (Fig. 7*I*), although CGS + QNP-induced chewing duration was short relative to that induced by the normal dose of QNP. The leading causes of this weak effect may be sedative effect of the combination treatment. While CGS alone and subthreshold dose of QNP did not cause sedation, CGS + QNP showed sedative effects, suggesting that CGS + QNP combination facilitated both QNP-induced chewing behavior and CGS-induced sedative effect (Barraco et al., 1994; Chen et al., 2001). Despite the lowered locomotion, CGS + QNP showed significant increase in chewing behavior and, indicating the involvement of A_2A_ receptor signaling in the CS on QNP-induced repetitive chewing behavior.

### Istradefylline rescued both the behavioral and cognitive symptoms in QNP-treated mice

**Figure 8.**
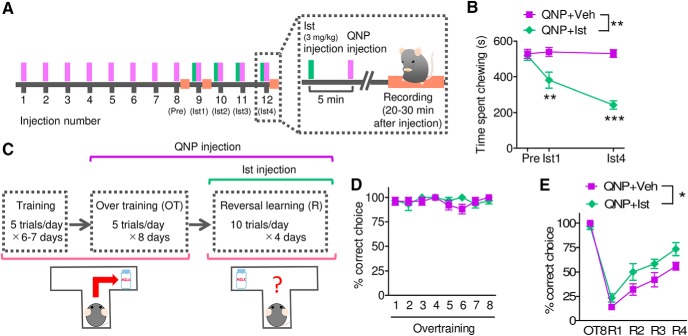
Istradefylline rescued both the behavioral and cognitive symptoms in QNP-treated mice. ***A***, Time course of recording of QNP-induced repetitive behavior combined with the short-term administration of istradefylline (Ist). ***B***, Time spent chewing during 20–30 min after QNP and Ist injections. [QNP+Veh; *n* = 5, QNP+Ist; *n* = 4, two-way repeated measures ANOVA; drug (*F*_(1,8.05)_ = 27.48, *p* = 0.0012), injection number (*F*_(1.15,9.26)_ = 17.45, *p* = 0.0025), interaction (*F*_(1.15,9.26)_ = 17.78, *p* = 0.0024), Bonferroni *post hoc* test; **p < 0.01, ***p < 0.001.] ***C***, Time course of a spatial discrimination task and a reversal learning test combined with the short-term administration of Ist. ***D***, ***E***, Percentage of correct choices during over training (***D***) and reversal learning (***E***). [QNP+Veh; *n* = 5, QNP+Ist; *n* = 6, ***D***: two-way repeated measures ANOVA; drug (*F*_(1,25.01)_ = 0.72, *p* = 0.4175), session number (*F*_(2.78,69.53)_ = 0.78, *p* = 0.5068), interaction (*F*_(2.78,69.53)_ = 1.04, *p* = 0.3878), ***E***: two-way repeated measures ANOVA; drug (*F*_(1,36)_ = 7.29, *p* = 0.0244), session number (*F*_(4,144)_ = 68.34, *p* < 0.0001), interaction (*F*_(4,144)_ = 1.58, *p* = 0.2010), *p < 0.05.]

The local injection experiments indicate the involvement of A_2A_ receptor and MEK/ERK signaling on QNP-induced behavioral abnormality, we assumed that an A_2A_ receptor antagonist could rescue QNP-induced chewing behaviors. A single administration of an A_2A_ receptor antagonist, istradefylline (3 mg/kg), significantly decreased the total chewing time in QNP-treated mice, and this effect was potentiated following repeated injections (Fig. 8*A*,*B*). In addition to the effect on the repetitive behavior, the short-term administration of istradefylline improved reversal learning in QNP-treated mice (Fig. 8*C–E*), suggesting the rapid effects of istradefylline on both SSRI-responsive and SSRI-resistant symptoms.

### Istradefylline rescued the altered synaptic plasticity in CS iMSNs from QNP-treated mice

**Figure 9.**
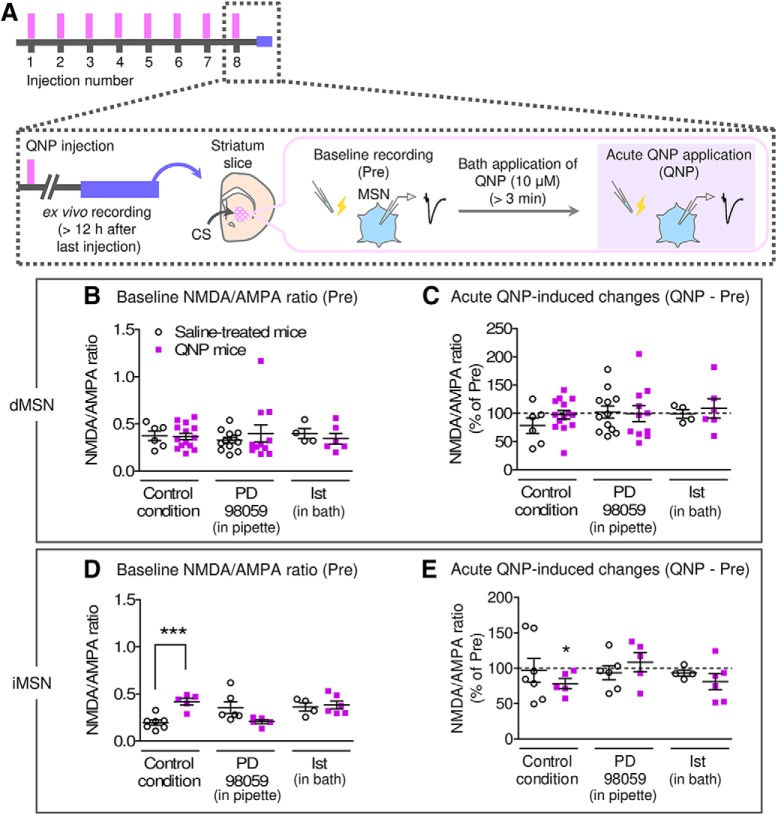
Altered synaptic functions in the CS iMSNs from QNP-treated mice was rescued by an A_2A_ antagonist. ***A***, Time course of electrophysiological recordings from CS MSNs. ***B***, Baseline NMDA/AMPA ratios recorded from CS dMSNs. [Control condition: saline; *n* = 6 from 5 mice, QNP; *n* = 14 from 5 mice, Student’s *t* test; *t*_(18)_ = 0.09770, *p* = 0.9233, PD98059: saline; *n* = 12 from 4 mice, QNP; *n* = 11 from 4 mice, unpaired *t* test with Welch’s correction; *t*_(12)_ = 0.7569, *p* = 0.4637, Ist (istradefylline): saline; *n* = 4 from 3 mice, QNP; *n* = 6 from 3 mice, Student’s *t* test; *t*_(8)_ = 0.6724, *p* = 0.5203.] ***C***, Bath application of QNP-induced changes in the NMDA/AMPA ratio recorded from CS dMSNs. (Control condition: saline; *n* = 6 from 5 mice, *t*_(5)_ = 1.587, *p* = 0.1735, QNP; *n* = 14 from 5 mice, *t*_(13)_ = 0.3219, *p* = 0.7526, PD98059: saline; *n* = 12 from 4 mice, *t*_(11)_ = 0.2176, *p* = 0.8317, QNP; *n* = 11 from 4 mice, *t*_(10)_ = 0.03232, *p* = 0.9748, Ist: saline; *n* = 4 from 3 mice, *t*_(3)_ = 0.1016, *p* = 0.9255, QNP; *n* = 6 from 3 mice, *t*_(5)_ = 0.5138, *p* = 0.6293. One sample *t* test compared with 100.) ***D***, Baseline NMDA/AMPA ratios recorded from CS iMSNs. (Control condition: saline; *n* = 7 from 3 mice, QNP; *n* = 5 from 4 mice, Student’s *t* test; *t*_(10)_ = 5.067, *p* = 0.0005, PD98059: saline; *n* = 6 from 4 mice, QNP; *n* = 5 from 3 mice, unpaired *t* test with Welch’s correction; *t*_(6)_ = 2.277, *p* = 0.0630, Ist: saline; *n* = 4 from 3 mice, QNP; *n* = 6 from 3 mice, Student’s *t* test; *t*_(8)_ = 0.3501, ***p = 0.7353.) ***E***, Bath application of QNP-induced changes in the NMDA/AMPA ratio recorded from CS iMSNs. (Control condition: saline; *n* = 7 from 3 mice, *t*_(6)_ = 0.1710, *p* = 0.8699, QNP; *n* = 5 from 4 mice, *t*_(4)_ = 3.019, *p = 0.0392, PD98059: saline; *n* = 6 from 4 mice, *t*_(5)_ = 0.6388, *p* = 0.5510, QNP; *n* = 5 from 3 mice, *t*_(4)_ = 0.6333, *p* = 0.5610, Ist: saline; *n* = 4 from 3 mice, *t*_(3)_ = 1.547, *p* = 0.2195, QNP; *n* = 6 from 3 mice, *t*_(5)_ = 1.684, *p* = 0.1529. One sample *t* test compared with 100.)

Rapid effects of istradefylline on SSRI-resilient chewing behaviors suggests a different action mechanism of istradefylline from that of chronic SSRI. Therefore, we tested the above-mentioned hypothesis that istradefylline acts on the CS iMSNs by using electrophysiological recordings (Fig. 9*A*).

First, to investigate repeated QNP injection-induced changes in the CS MSNs, we recorded the basal NMDA/AMPA ratios of the CS MSNs from mice received repeated QNP or saline injection. Compared to the saline-treated mice, the basal NMDA/AMPA ratio was increased in iMSNs from QNP-treated mice, whereas no significant changes were observed in dMSNs (Fig. 9*B*,*C*). In addition, to examine the effects of challenge with QNP, we compared NMDA/AMPA ratios before (basal) and after the bath application of QNP (10 μM), which mimics an *in vivo* challenge with QNP. In contrast to the basal NMDA/AMPA ratio, the bath application of QNP (10 μM) induced a significant reduction in the NMDA/AMPA ratio in iMSNs from QNP-treated mice but not from saline-treated mice (Fig. 9*D*,*E*). The intracellular application of PD98059 (50 μM) through the recording electrode restored those abnormal synaptic functions for both baseline and QNP application induced-responses (Fig. 9*C*,*E*), suggesting that, in QNP-treated mice, CS iMSNs showed altered synaptic plasticity through D_2_-ERK signaling.

We then examined the effects of istradefylline on the QNP-induced synaptic changes. As expected, the bath application of istradefylline (10 μM) had no effects on the NMDA/AMPA ratios recorded from the CS dMSNs (Fig. 9*B*,*C*). In case of iMSNs, bath application of istradefylline tend to increase the baseline NMDA/AMPA ratio from saline-treated mice and no further increase was observed in QNP-treated mice (Fig. 9*D*). The QNP application induced-response in iMSNs from QNP-treated mice was also inhibited by bath application of istradefylline (Fig. 9*E*).

## Discussion

In this study, we characterized QNP sensitization-induced OCD-related behaviors and treatment responses in mice. Both the cognitive inflexibility and the abnormal lOFC activity in QNP-treated mice were rescued by chronic high-dose SSRI, whereas these treatments failed to improve the repetitive chewing behavior. Finally, we showed that D_2_ signaling in CS iMSNs, where lOFC neurons project, was required for this repetitive behavior and that the short-term administration of a clinically approved A_2A_ antagonist, istradefylline, rescued both SSRI-responsive and SSRI-resistant symptoms in QNP-treated mice.

As OCD-like behaviors in rodents, most existing reports evaluate repetitive behaviors (e.g., excessive grooming) and perseverative behaviors (e.g., deficits in spontaneous alternation and reversal learning; Alonso et al., 2015; Stuchlik et al., 2016). However, the pathophysiological mechanisms of those OCD-like behaviors are not fully understood, especially about the differences in the neurologic and therapeutic mechanisms between the two symptoms (Alonso et al., 2015; Stuchlik et al., 2016). QNP-treated mice exhibited both of these behaviors, demonstrating that the QNP-induced behavioral abnormalities in mice are convenient for examining pathophysiological mechanisms and subsequent drug screening.

Dopaminergic drug-induced repetitive behaviors are widely reported both in basic and clinical studies. In animal experiments, single administration of psychostimulant elicits various stereotypic behaviors, including chewing and grooming (Izawa et al., 2006; Milesi-Hallé et al., 2007). Recent evidence demonstrated the modulating effects of mutation of OCD-related gene in psychostimulant-induced stereotypy (Zike et al., 2017). In psychostimulant abusers and parkinsonian patient with dopaminergic replacement therapy, non-goal-directed complex stereotypies, which are similar to OCD symptoms, are observed (Voon et al., 2009; Fasano and Petrovic, 2010). These observations suggest the existence of the common mechanisms underlying OCD and dopaminergic drug-induced stereotypies. Based on this, QNP-induced repetitive chewing might also share the common mechanisms, while further discrimination against other repetitive behaviors, such as tic disorder which is often comorbid with OCD, should be carefully performed.

SSRIs are the first-choice drugs for OCD patients, and in SSRI-responsive patients, SSRIs normalizes the activity of the anterior lOFC (Saxena et al., 1999). The OFC can be divided into the medial OFC (mOFC) and the lOFC, which encode essentially similar but distinct information (Milad and Rauch, 2012). For instance, the mOFC is activated by positive reward stimuli, while the lOFC responds to punishment (Ursu and Carter, 2005; Plassmann et al., 2008). OCD patients exhibit a deficiency in punishment-related learning (Nielen et al., 2009), and possibly as a result of this abnormal punishment processing, OCD patients, especially in severe cases, are unable to stop their compulsions, even when the compulsions cause a disadvantage to the patients themselves (Pauls et al., 2014). In this situation, lOFC hyperactivity might be one of the common neurologic bases and a therapeutic target for cognitive inflexibility among OCD patients and QNP-treated mice.

In contrast to cognitive inflexibility, repetitive chewing behavior was SSRI-resistant, suggesting that the inhibition of lOFC outputs was insufficient to improve repetitive behavior. Recent optogenetic research demonstrated that the repeated activation of the OFC-striatum pathway increased grooming behavior (Ahmari et al., 2013), and the overall inhibition of the OFC-striatum pathway contributed to the inhibition of compulsive grooming behavior in a genetic OCD model, the *Dlgap3* (*Sapap3*)-knock-out mice (Burguière et al., 2013). However, both in the optogenetic stimulation model and *Sapap3*-knock-out mice, repetitive behavior was reduced by SSRI administration (Welch et al., 2007; Burguière et al., 2013). One possible reason for this discrepancy is that the input-output balance between striatal dMSNs and iMSNs was differentially altered in QNP-treated mice and the overall decrease in lOFC inputs failed to rectify the abnormal activity balance between MSNs subtypes. In other word, after challenge with QNP, the OFC-striatum iMSN pathway rather than the OFC-striatum dMSN pathway was potentiated, causing the abnormal transmission of OFC information. While additional experiments on the pathway-specific control are required, hyperactivity in the lOFC-CS iMSN pathway might contribute to SSRI-resistant repetitive behavior in QNP-treated mice.

Changes in the activity balance between iMSNs and dMSNs contributes to habit formation (Shan et al., 2015; O'Hare et al., 2016). Recent research demonstrated that the selective reduction of excitatory inputs from the OFC to striatal dMSNs promotes habit formation (Renteria et al., 2018). Considering that habit learning is facilitated in OCD patients (Gillan et al., 2011, 2016), this finding supports the idea that an abnormal activity balance between iMSNs and dMSNs contributes to OCD pathophysiology. Considering that the OFC-striatum pathway contributes to goal-directed behavior, and the activation of the OFC promotes a goal-directed behavioral pattern rather than habit formation (Gremel and Costa, 2013; Gourley and Taylor, 2016), an abnormal activity balance between iMSNs and dMSNs might explain the clinical features of OCD.

Although the D_2_ receptor signal theoretically inhibits neurons, a recent study demonstrated that the acute activation of D_2_ receptors does not inhibit iMSNs (Lemos et al., 2016), possibly because of the lack or low levels of expression of G protein-coupled inwardly rectifying potassium channels in iMSNs (Kobayashi et al., 1995). The sustained activation of D_2_ receptors by a selective agonist activates ERK signaling in iMSNs, possibly through D_2_ receptor internalization (Brami-Cherrier et al., 2002; Shioda et al., 2017), resulting in the activation of rather than the inhibition of iMSNs. Supporting this idea, both in OCD patients and in QNP-sensitized rats after challenge with QNP, D_2_ receptor occupancy was decreased (Denys et al., 2013; Servaes et al., 2017), suggesting increased the D_2_ receptor signaling (e.g., increased baseline dopamine release) and/or facilitation of D_2_ receptor internalization. Accordingly, repeated QNP injection might mimic the abnormal D_2_ receptor signaling, resulting in activation of iMSNs.

In iMSNs, the A_2A_ receptor signal contributes to synaptic plasticity (Shen et al., 2008). In contrast, the blockade of A_2A_ receptors by istradefylline inhibits iMSNs (Shen et al., 2008) and then facilitates dMSN-mediated signal transduction. A_2A_ receptor signaling in the striatum is involved in the mediation of goal-directed learning and habit formation in naïve mice (Yu et al., 2009; Li et al., 2016), supporting our findings that istradefylline improved the cognitive inflexibility in QNP-treated mice.

Besides cognitive inflexibility, istradefylline improved the SSRI-resistant repetitive chewing behavior, suggesting the therapeutic potential of A_2A_ antagonists for a wide range of OCD symptoms. Future work is needed to determine whether istradefylline and other A_2A_ antagonists show similar therapeutic effects in other OCD-related behaviors and model animals.

Recent evidence suggests that A_2A_ receptors and D_2_ receptors form heteromers and that a change in the surface expression of this heteromer might be involved in habit formation (He et al., 2016). In A_2A_-D_2_ heteromers, A_2A_ receptor signaling positively modulates D_2_ agonist-induced internalization and the resulting intracellular signaling, such as ERK phosphorylation (Borroto-Escuela et al., 2011; Huang et al., 2013). In accordance with this, co-stimulation of A_2A_ receptors and D_2_ receptors facilitated repetitive chewing behavior in QNP-sensitized mice. The A_2A_ signaling-mediated internalization of A_2A_-D_2_ heteromers might be involved in OCD pathophysiology and the anti-OCD action of istradefylline; however, further studies are required.

In conclusion, we characterized distinct treatment responses of OCD-related abnormalities in QNP-treated mice. Chronic high-dose SSRI rescued lOFC hyperactivity, while the therapeutic effect was restricted. An A_2A_ antagonist, istradefylline, normalized synaptic functions in CS iMSNs and improved both the SSRI-responsive and SSRI-resistant OCD-related behaviors in QNP-treated mice. Considering that istradefylline has already been approved as an anti-Parkinsonian agent, the present results support the drug repositioning of istradefylline to be a rapid-acting and effective anti-OCD drug.

## Synthesis

Reviewing Editor: Lorna Role, Stony Brook Decisions are customarily a result of the Reviewing Editor and the peer reviewers coming together and discussing their recommendations until a consensus is reached. When revisions are invited, a fact-based synthesis statement explaining their decision and outlining what is needed to prepare a revision will be listed below. The following reviewer(s) agreed to reveal their identity: Eleanor Simpson.

The reviewers are largely satisfied with the revisions made but ask that the results of the additional experiments conducted be included in the manuscript, despite the weak effect of CGS+QNP compared to the QNP-induced.
